# Self‐calibrated B_1_ ^+^ and B_0_ Field Inhomogeneities Estimation (SAFE) in 3D Magnetic Resonance Fingerprinting

**DOI:** 10.1002/mrm.70525

**Published:** 2026-07-21

**Authors:** Mengze Gao, Xiaozhi Cao, Daniel Raz Abraham, Zihan Zhou, Nan Wang, Congyu Liao, Yang Yang, Hanlin Sun, Hongwei Li, Yan Bai, Yu Shen, Yaping Wu, Meiyun Wang, Kawin Setsompop

**Affiliations:** ^1^ Department of Radiology Stanford University Stanford California USA; ^2^ Department of Electrical Engineering Stanford University Stanford California USA; ^3^ Department of Radiology University of California San Francisco San Francisco California USA; ^4^ Department of Radiology The Third Hospital of Mianyang Mianyang Sichuan China; ^5^ Sichuan Mental Health Center Mianyang Sichuan China; ^6^ Department of Radiology Henan Provincial People's Hospital Zhengzhou Henan China; ^7^ People's Hospital of Zhengzhou University Zhengzhou Henan China

**Keywords:** deep learning, field inhomogeneity, MR fingerprinting, quantitative MRI

## Abstract

**Purpose:**

To develop SAFE, a self‐calibrated framework to estimate B_1_^+^ and B_0_ field inhomogeneities directly from conventional magnetic resonance fingerprinting (MRF) acquisitions and improve its quantification accuracy.

**Methods:**

SAFE utilized a two‐step approach. First, two physics‐informed image markers are extracted from the MRF data to create a magnitude and a phase image that are highly correlated with B_1_^+^ and B_0_ inhomogeneities, respectively. Second, a deep learning (DL) network is applied to map these markers to quantitative field maps. SAFE was tested on 3D Spiral Projection Imaging MRF brain acquisition at 3T, where the network was trained and validated across a multi‐site, multi‐vendor dataset (*N* = 358) from healthy volunteers and clinical population. The capability of SAFE to be applied to unseen MRF sequences with different signal preparations and flip‐angle trains through adaptively retraining without the need for additional training data was also tested.

**Results:**

SAFE achieved normalized‐root‐mean‐square‐error within 3% against gold‐standard field‐calibration scans on both B_1_^+^ and B_0_ maps (*N* = 32), with high performance remaining on datasets from scanners that the training data were acquired from. T1 and T2 biases were shown to be effectively corrected on healthy volunteers, a patient with brain tumor, and a pediatric subject. Tissue quantification accuracy after SAFE‐estimated field correction was validated on a large patient cohort (*N* = 86). SAFE was also demonstrated to be adaptable to different MRF sequences without acquiring additional training data.

**Conclusion:**

By combining physics‐informed image markers with DL, the proposed SAFE framework enables calibration‐free estimation of B_1_^+^ and B_0_ field inhomogeneities at 3T for whole brain MRF.

## 1 Introduction

Magnetic resonance fingerprinting [1] (MRF) enables fast quantitative imaging. However, accurate quantification necessitates the characterization and incorporation of B_1_^+^ and B_0_ field inhomogeneities into the image reconstruction and parameter fitting process to avoid spatial bias and blurring artifacts [2, 3]. Although separate calibration scans can characterize these imperfections, such procedures prolong scan time, complicate workflow, and often require co‐registration. While fast calibration methods have been developed [4, 5], estimating inhomogeneities directly from MRF data remains desirable to obviate the need for additional acquisitions.

Along this theme, tailored MRF acquisition approaches [6, 7] have been developed to jointly estimate field imperfections and tissue parameters by enhancing MRF's signal sensitivity to field imperfections. Nevertheless, conventional MRF sequences, including those optimized via Cramer‐Rao Bound [8, 9], do not contain sufficient independent sensitivity to these field imperfections, where, for example, a change in its signal time‐course could be attributed either to a change in B_1_^+^ or a change in T2. Consequently, the joint fitting process can become ill‐posed, leading to poor field estimation and biased tissue parameter maps.

Traditional techniques such as autofocus [10] utilize phase information to estimate B_0_ through iterative searching; however, it might fail in regions where B_0_ or its variations are large. Recently, deep learning (DL) approaches have emerged as data‐driven alternatives. However, many approaches are based on identifying blurring artifacts for B_0_ estimation [11], which can be difficult to decouple from complex anatomical variability. To address these limitations, in this work, a tailored DL approach is developed to accurately and robustly estimate B_1_^+^ and B_0_ inhomogeneities directly from conventional MRF data. Accordingly, the self‐calibrated B_1_^+^ and B_0_ field estimation (SAFE) for MRF is proposed. With SAFE, two physics‐informed image markers are extracted from the MRF data to form a magnitude and a phase image marker that are highly correlated only with B_1_^+^/B_0_ inhomogeneities, respectively. These markers are then mapped to field map estimates via a DL network. This strategy circumvents the ill‐posedness of the aforementioned joint estimation and ensures robust performance by providing the network with physics‐guided inputs. As a proof of concept, SAFE was implemented on a 3D Fast imaging with steady‐state precession (FISP) MRF acquisition [12, 13] with 3D spiral projection imaging (3D‐SPI) readout for whole brain imaging at 3T, where the network was trained on diverse multi‐vendor dataset. The model was demonstrated to provide accurate B_1_^+^ and B_0_ field maps. Furthermore, a novel sequence‐adaptive retraining scheme was validated, allowing the application of SAFE to new, unseen MRF sequences without reacquiring new training data.

Source code along with an example set of paired MRF data with B_1_ ^+^ and B_0_ maps from calibration scans across 20 subjects are publicly available at https://github.com/oagmz/SAFE.git.

## 2 Methods

### 2.1 Background on Data Acquisition, Image Reconstruction, B_1_ ^+^ and B_0_ Inhomogeneities and Their Corrections

The SAFE framework was developed and evaluated on a FISP‐MRF sequence [12], wherein 500 repetition times (TRs) with varying FAs (Figure 1A) were acquired following an adiabatic inversion pulse. A tiny‐golden‐angle‐shuffling (TGAS) 3D‐SPI readout [13] was utilized for data acquisition (Figure 1B), and spatiotemporal subspace reconstruction [14, 15] was employed using five temporal basis [16].

(1) $$\hat{U}=\arg\min_{U}\;\sum_{c=1}^{N_{c}}{\left\|\Omega FS_{C}U{\hat{V}}^{H}-y_{c}\right\|}_{2}^{2}+\lambda R\left(U\right)$$

where $\hat{U}$ denotes the estimated subspace coefficients; $y_{c}$ the k‐space measurement of the c th coil; $N_{c}$ the number of receive coils; $S_{C}$ the coil sensitivity map; F the nonuniform fast Fourier transform (NUFFT) operator; Ω the k‐space undersampling mask; ${\hat{V}}^{H}$ the temporal basis vectors; and R(·) the Locally‐Low‐Rank operator with the regularization parameter λ. Quantitative maps were subsequently estimated by matching subspace coefficients to a pre‐calculated dictionary.

**FIGURE 1:**
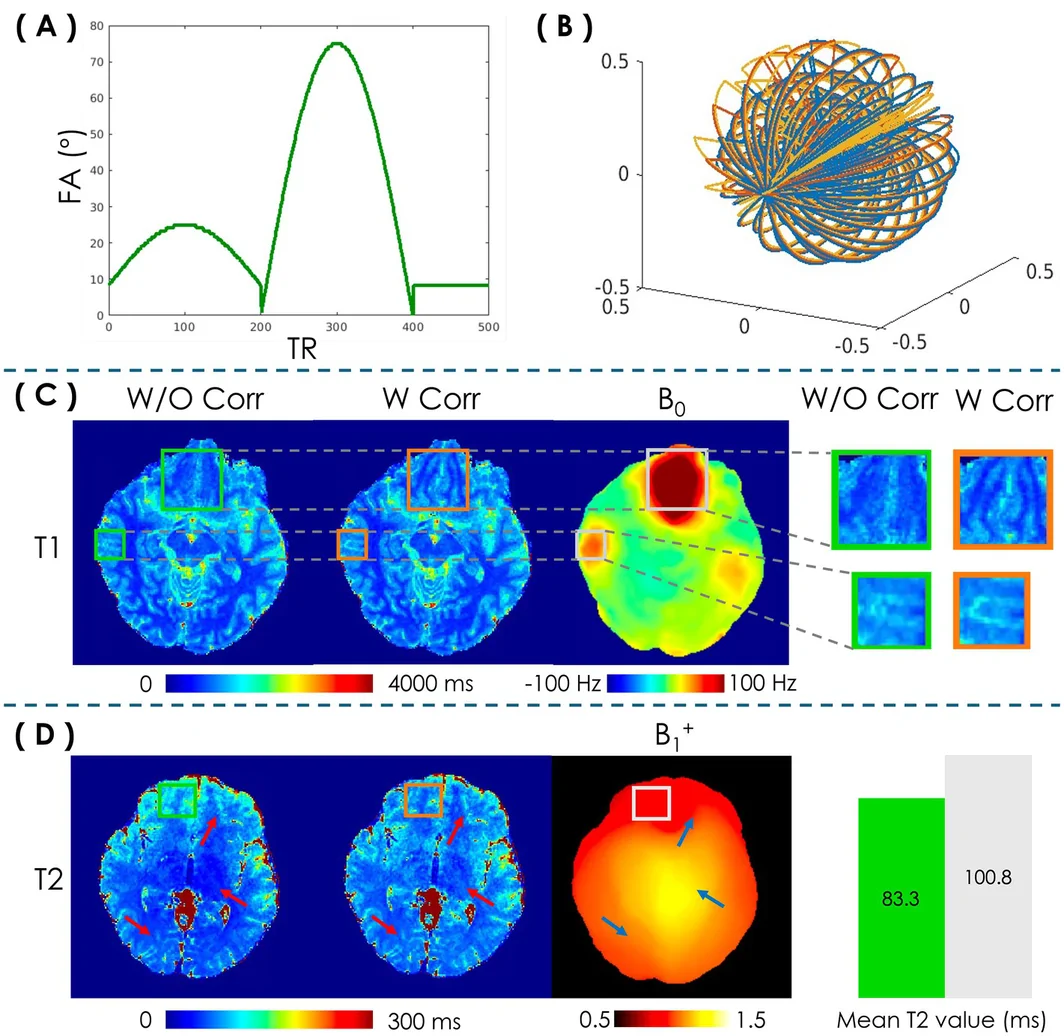
Overview of the 3D FISP‐MRF sequence and the effects of field inhomogeneities. (A) The flip‐angle (FA) train used in the sequence. (B) An example of the tiny‐golden‐angle‐shuffling (TGAS) 3D spiral projection imaging (SPI) trajectory. (C) B_0_ inhomogeneity induced blurring artifacts in the T1 map, particularly in areas of large inhomogeneities of the temporal lobes and frontal lobes (zoomed in regions). To correct for this, a B_0_ map along with multi‐frequency interpolation (MFI) can be incorporated into the MRF reconstruction to achieve improved T1 map (W Corr). (D) B_1_^+^ inhomogeneity caused spatial shading artifacts in the T2 map, leading to 21.5% bias in the boxed region, that can be corrected (W Corr) by incorporating the spatial flip‐angle variability into the dictionary fitting process.

During the RF excitation, B_1_^+^ inhomogeneity, arising from coil design as well as patient‐specific dielectric properties [17], induces spatial variability in the MRF FA train that deviates from the prescribed one, potentially causing spatially varying bias in the T1 and T2 estimates. In typical MRF sequences, T1 sensitivity is derived primarily from data acquired subsequent to the initial B_1_^+^‐insensitive adiabatic inversion pulse, while T2 sensitivity is mainly obtained from the subsequent varying FA train [9]. Thus, B_1_^+^ inhomogeneity predominantly biases the T2 map (Figure 1D). Signal evolutions under different levels of B_1_^+^ variabilities can be simulated and incorporated into the MRF dictionary and used in a joint field imperfections and tissue parameters fitting process. However, such joint fitting can become ill‐posed, yielding biased tissue parameter maps and poor B_1_^+^ estimation (see Figure 2B, 1^st^ and 3^rd^ column, to compare flip‐angle map estimated from joint fitting vs. from B_1_^+^ calibration scan). Alternatively, utilizing a B_1_^+^ map from calibration scan as prior significantly improves the fitting condition [16].

**FIGURE 2:**
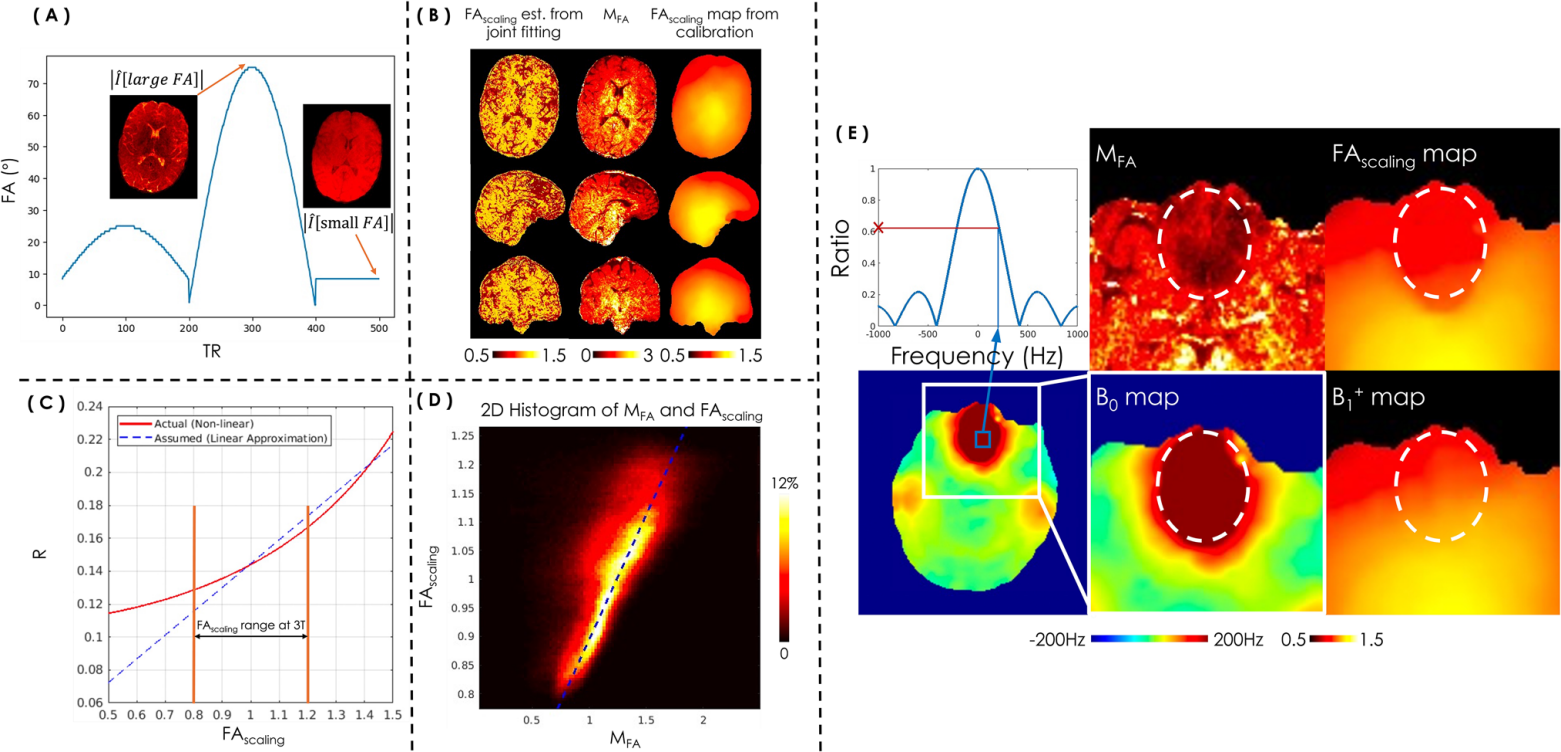
Generation of physics‐informed image markers M_FA_ for FA_scaling_ map estimation. (A) Time‐resolved images corresponding to the time points (TRs) with large and small nominal FAs were used to generate the FA_scaling_‐sensitive marker, M_FA_. (B) Visual comparison between (i) estimated FA_scaling_ map from a joint field‐map and tissue parameters dictionary fitting, (ii) proposed $M_{\mathrm{FA}}$, and (iii) ground truth FA_scaling_ map obtained from reference field‐calibration scans. The estimated FA_scaling_ map from the joint fitting contains large biases and artifacts whereas the proposed image marker, M_FA_, exhibits higher correlation with the ground truth FA_scaling_ map. (C) Numerical simulation of the theoretical ratio R as a function of FA_scaling_ with ${\mathrm{FA}}_{\mathrm{low}}=8^{{}^{\circ}}$ and ${\mathrm{FA}}_{\text{high}}=75^{{}^{\circ}}$ in our implementation. (D) The 2D histogram showing the linear correlation between M_FA_ and ground truth FA_scaling_ (FA_scaling_ $=0.44\cdot M_{\mathrm{FA}}+0.46,R^{2}=0.93$). (E) Top‐left panel shows the limited RF excitation bandwidth of the water‐only *rect* excitation pulse employed in our MRF sequence (x‐axis:−1000 Hz˜1000 Hz, y‐axis: 0˜1). In a large susceptibility region at the frontal sinus, B_0_ inhomogeneity noticeably reduces the effective FA, where the effective spatial FA_scaling_ is a multiplicative combination of B_1_^+^ inhomogeneity and this B_0_‐induced flip‐angle reduction. The FA_scaling_‐sensitive marker, M_FA_, correlates well with this effective FA_scaling_ map.

During the spiral readout, B_0_ inhomogeneity, influenced by magnet imperfections and patient‐induced susceptibility variations [18], causes additional spatiotemporal phase modulation, resulting in image blurring (Figure 1C). To correct this, a B_0_ map along with multi‐frequency interpolation [19] (MFI) can be incorporated into the subspace reconstruction. For 3T brain imaging targeted in this work, five demodulation frequencies $\omega_{m}$ = [−200:100:200] Hz were used.

Beyond their individual effects, the interaction between B_1_^+^ and B_0_ inhomogeneity is also worth noting. Since typical RF excitation in MRF is bandwidth‐limited, particularly with water‐only excitation [16], B_0_ inhomogeneity noticeably reduces the effective FA, where the effective spatial FA_scaling_ is a multiplicative combination of B_1_^+^ inhomogeneity and this B_0_‐induced flip‐angle reduction [4]. This effective FA_scaling_ can then be accounted for during the dictionary fitting described above.

### 2.2 The SAFE Approach

The MRF signal evolution is simultaneously sensitive to tissue properties as well as spatial variability in FA‐scaling (FA_scaling_) and B_0_ inhomogeneity. The aim of SAFE is to create image markers to isolate part of the acquired MRF signal that is mainly correlated to either FA_scaling_ variability or B_0_ inhomogeneity and utilize a DL model to estimate FA_scaling_ map and B_0_ map robustly from these image markers. Note that if desired, B_1_^+^ map can be further extracted from the estimated FA_scaling_ map and B_0_ map by dividing out the B_0_‐induced FA_scaling_ effect.

### 2.3 Image Marker for FA_scaling_ Estimation

From the reconstructed MRF subspace coefficient, time‐resolved images $\hat{I}$ could be extracted,

(2) $$\hat{I}=\hat{U}{\hat{V}}^{H}$$

A large component of the MRF signal at a given *TR* comes from signal generated by the RF excitation at that *TR*: $\sin\;(\text{FAscaling}\left(r\right)\cdot {\mathrm{FA}}_{\text{nominal}}(\mathrm{TR}))$. At a low ${\mathrm{FA}}_{\text{nominal}}$ (e.g., < 10°):

(3) $$\sin\;\left({\mathrm{FA}}_{\text{scaling}}\left(r\right)\cdot {\mathrm{FA}}_{\mathrm{low}}\right)\approx {\mathrm{FA}}_{\text{scaling}}\left(r\right)\cdot {\mathrm{FA}}_{\mathrm{low}}$$

whereas for a large ${\mathrm{FA}}_{\text{nominal}}$ (e.g., > 70°),

(4) $$\sin\;\left({\mathrm{FA}}_{\text{scaling}}\left(r\right)\cdot {\mathrm{FA}}_{\text{high}}\right)\approx \sin\left({\mathrm{FA}}_{\text{high}}\right)$$

To isolate FA_scaling_(*r*)‐related features, we defined a theoretical ratio,

(5) $$R=\frac{\;\sin\;\left({\mathrm{FA}}_{\text{scaling}}\left(r\right)\cdot {\mathrm{FA}}_{\mathrm{low}}\right)}{\sin\;\left({\mathrm{FA}}_{\text{scaling}}\left(r\right)\cdot {\mathrm{FA}}_{\text{high}}\right)}\approx \frac{{\mathrm{FA}}_{\mathrm{low}}}{\sin\left({\mathrm{FA}}_{\text{high}}\right)}\cdot {\mathrm{FA}}_{\text{scaling}}\left(r\right)$$

Taking the ratio of the time‐resolved reconstructed images at such two time‐points acts to remove common image scaling from, for example, coil sensitivity and proton density, and provides a surrogate image marker sensitive mainly to FA_scaling_(*r*), termed M_FA_. In our implementation, images of time‐points with 8° and 75° nominal FAs were chosen to form M_FA_ (Figure 2A).

(6) $$M_{\mathrm{FA}}=\frac{\left|\hat{I}\left[FA_{\text{nominal}}=8^{{}^{\circ}}\right]\right|}{\left|\hat{I}\left[FA_{\text{nominal}}=75^{{}^{\circ}}\right]\right|}$$

As simulated in (Figure 2C), R exhibits a strictly monotonic relation with FA_scaling_, and has small approximation error under linear assumption. The 2D histogram shown in (Figure 2D) confirms that $M_{\mathrm{FA}}$ followed the linear trend with FA_scaling_, providing a robust prior for field estimation. Figure 2E shows a significant B_0_‐induced FA_scaling_ effect in a large susceptibility variation region for an MRF acquisition performed using a water‐only excitation *rect* pulse. Here, M_FA_ correlates well with the FA_scaling_ map that contains the multiplicative effect of B_1_^+^ inhomogeneity and B_0_‐induced flip‐angle reduction.

In addition to the signal contribution generated by the current RF excitation, the overall image at a particular TR is also affected by the excitation spin‐history and signal refocusing, which are dependent on the tissue properties. For tissues with long T2s (e.g., cerebrospinal fluid), significant additional stimulated echo contributes to the overall signal at the large FA time‐point, as observed in the cerebrospinal fluid regions of M_FA_ in (Figure 2B). These complex, non‐linear dependencies make the M_FA_‐to‐FA_scaling_ mapping deviate from the ideal theoretical curve. This is the motivation for employing a DL algorithm to implicitly resolve these tissue‐dependent biases from underlying anatomy and provide accurate FA_scaling_ estimates.

### 2.4 Image Marker for B_0_ Estimation

SAFE leverages the phase information of the reconstructed images for robust B_0_ estimation. A common phase‐based B_0_ estimation approach is to estimate B_0_ using image phase differences at multiple echo times (TEs). In the MRF acquisition, data is typically collected at a single TE, but an average‐contrast image at a second TE can be formed by combining data of the readout rewinders across different TRs (Figure 3A,B). The phase difference of the two average‐contrast images at different TEs can then be used to form the image marker, M_B0_:

(7) $$M_{B0}=\frac{\text{Angle}\left({\hat{I}}_{\text{out}}\cdot \text{conj}\left({\hat{I}}_{\text{in}}\right)\right)}{\Delta \mathrm{TE}}\approx 2\pi \cdot B_{0}$$

where ${\hat{I}}_{\text{out}}$ denotes the average‐contrast image obtained from the spiral readouts; ${\hat{I}}_{\text{in}}$ the average‐contrast image from the rewinders; conj(·) the conjugate operator; and ΔTE the echo time difference between the spiral readout and the rewinder.

**FIGURE 3:**
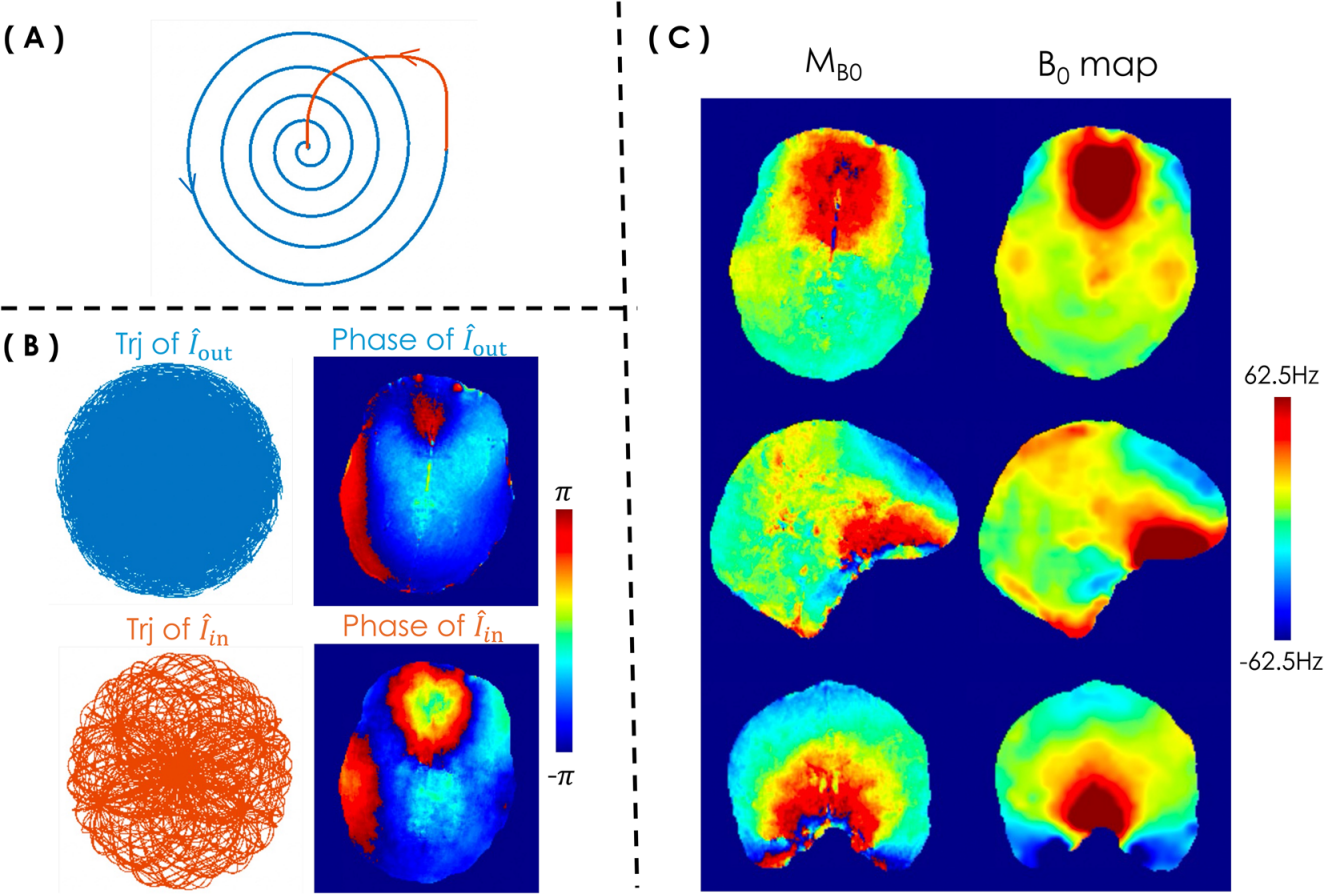
Generation of physics‐informed image markers M_B0_ for B_0_ map estimation. (A) K‐space trajectory for a single spiral interleave, showing the readout (blue) and rewinder (orange) segments. (B) 3D K‐space trajectory and resulting phase images of ${\hat{I}}_{\text{out}}$ and ${\hat{I}}_{\text{in}}$, which were generated by combining the readout segments (blue in A) and the rewinder segments (orange in A) across all the TRs, respectively. The phase difference between these two images formed the B_0_‐sensitive marker, M_B0_. (C) Visual comparison showing high spatial correlation between M_B0_ and the ground truth B_0_ map.

Figure 3 C indicates that the M_B0_ contains high similarity to the B_0_ map obtained from a separate field‐calibration scan, but with a certain level of phase wrapping due to the relatively long ΔTE (8 ms), and incoherent aliasing artifacts caused by the higher undersampling rate of the rewinder trajectory. These nonlinear imperfections necessitate a subsequent DL algorithm to resolve by leveraging strong prior knowledge of the expected spatial smoothness of the B_0_ maps.

### 2.5 SAFE Network Architecture and Training

Two DL networks were trained with input M_FA_ or M_B0_ respectively and output corresponding FA_scaling_ or B_0_ map. Both networks utilized a U‐Net [20] structure with skip connections removed (Figure S1). During each encoding block, volume size decreased by a factor of 8 while channel size expanded by a factor of 2; the decoding path performed the reverse operations. Each convolutional layer employed a 3‐by‐3‐by‐3 kernel, followed by instance normalization and LeakyReLU activation. A final convolution layer with a 5‐by‐5‐by‐5 kernel was used to enforce spatial smoothness. Training deployed the Adam optimizer and L1 loss, calculated within a high confidence masked region (via BET [21]).

### 2.6 Sequence‐Agnostic Adaption

The DL network training requires matching input and output pairs, which are sequence specific. As such, the model weights trained on one MRF sequence can't be directly applied to another. A wide variety of MRF sequences have been developed, so it would be helpful to be able to adapt the training dataset obtained for one sequence for use to train the SAFE‐networks of any other MRF sequences without having to reacquire new training data.

Herein a sequence adaptive scheme, Adaptive‐SAFE, is developed (Figure S2), where the B_1_^+^, B_0_, PD, T1, and T2 maps, extracted from paired MRF and field‐calibration scan of the original MRF sequence, are used to simulate the target sequence's signal evolution based on its sequence parameters. A synthetic image marker, M_FA_new_, can then be extracted from this simulated data and paired with the original FA_scaling_ map to retrain the FA_scaling_ estimation network. If the target sequence has the same readout trajectory, it's unnecessary to retrain the B_0_ estimation network. But if this isn't the case, simulation of the MRF forward model is necessary including the new trajectory and B_0_ map, so that ${\hat{I}}_{\text{out}\_\mathrm{new}}$ and ${\hat{I}}_{\text{in}\_\mathrm{new}}$ can be obtained to create M_B0_new_, which can then be paired with the original B_0_ map to retrain the B_0_ estimation network.

In this work, Adaptive‐SAFE was tested on two new target sequences. The first is a FISP‐MRF sequence that utilizes 1000 TRs compared to our original MRF sequence (500 TRs), while also employing markedly different FA train that we adopted from Figure 1B in Jiang et al. [12] In the subsequent sections, we will refer to this sequence as “1000TRs‐MRF.” The second is the ViSTa‐MRF [22] (Visualization of Short Transverse relaxation time component MRF) sequence which has been designed for quantitative myelin water fraction mapping. To sensitize its acquisition to myelin water, ViSTa‐MRF contains a double inversion preparation module as well as markedly different flip‐angle train over 660 TRs. In this work, we focused on testing the adaptability property of SAFE for FA_scaling_, thus, the same 3D‐SPI readout is employed for both sequences and only the paired synthesized FA_scaling_ datasets from the proposed sequence adaptive scheme were used to retrain the FA_scaling_ estimation network respectively.

### 2.7 Data Acquisition

All data were acquired on 3T GE and Siemens scanners with approval of the institutional review board with informed consent obtained.

3D FISP‐MRF: Data were acquired at 1 mm isotropic resolution over the whole brain using 3D‐SPI acquisition with TGAS readouts. The sequence used a 1.7 ms *rect* pulse for water‐selective excitation, a 500‐TR FA train, over a 2‐min scan as per [16]. The readout duration was 6.7 ms, followed by a 1.3 ms rewinder.

1000TRs‐MRF: Data were acquired at 1 mm isotropic resolution with 3D TGAS‐SPI readout, with a different FA train over 1000 TRs, resulting in a 6‐min scan.

ViSTa‐MRF: Data were acquired at 1 mm isotropic resolution with 3D TGAS‐SPI readout, featuring a Cramér–Rao lower bound optimized FA train over 660 TRs, resulting in a 5‐min scan [22].

Ground truth field maps: Gold standard B_1_^+^ and B_0_ maps were acquired using a Bloch‐Siegert pulse sequence [5] and a dual‐echo gradient echo sequence respectively, using efficient 3D spiral projection readout [4]. Both were acquired with 4 mm isotropic resolution and a total scan time of 16 s and were subsequently registered to the MRF dataset. The FA_scaling_ maps were calculated from the B_1_^+^ and B_0_ maps based on the spectral response of the excitation pulse (Figure 2E top‐left panel).

A SAFE model was first trained on 358 3D‐FISP MRF datasets from adult healthy volunteers (*N* = 137) and patients (*N* = 221) acquired from 6 scanner models across 4 institutions (Stanford University, UCSF, Henan People's Hospital, and Third Hospital of Mianyang; see Table S1) and tested on subjects across 8 different GE and Siemens scanner models, consisting of 5 subjects from each of the 6 scanners used in acquiring training data, and 1 subject from each of 2 additional scanners to demonstrate generalizability.

The 1000TRs‐MRF and ViSTa‐MRF Adaptive‐SAFE models for FA_scaling_ map estimation were retrained separately on 20 respective synthesized 3D datasets (generated from original sequence's training data) and tested on 3 datasets from healthy volunteers for each sequence.

## 3 Results

SAFE's performance across diverse scanner types was evaluated. Figure 4 shows the estimation result on the test cases. SAFE's performance was further evaluated across various populations (healthy volunteers, a patient with brain tumor, and a pediatric patient) on field estimation and tissue parameter maps before and after field correction.

**FIGURE 4:**
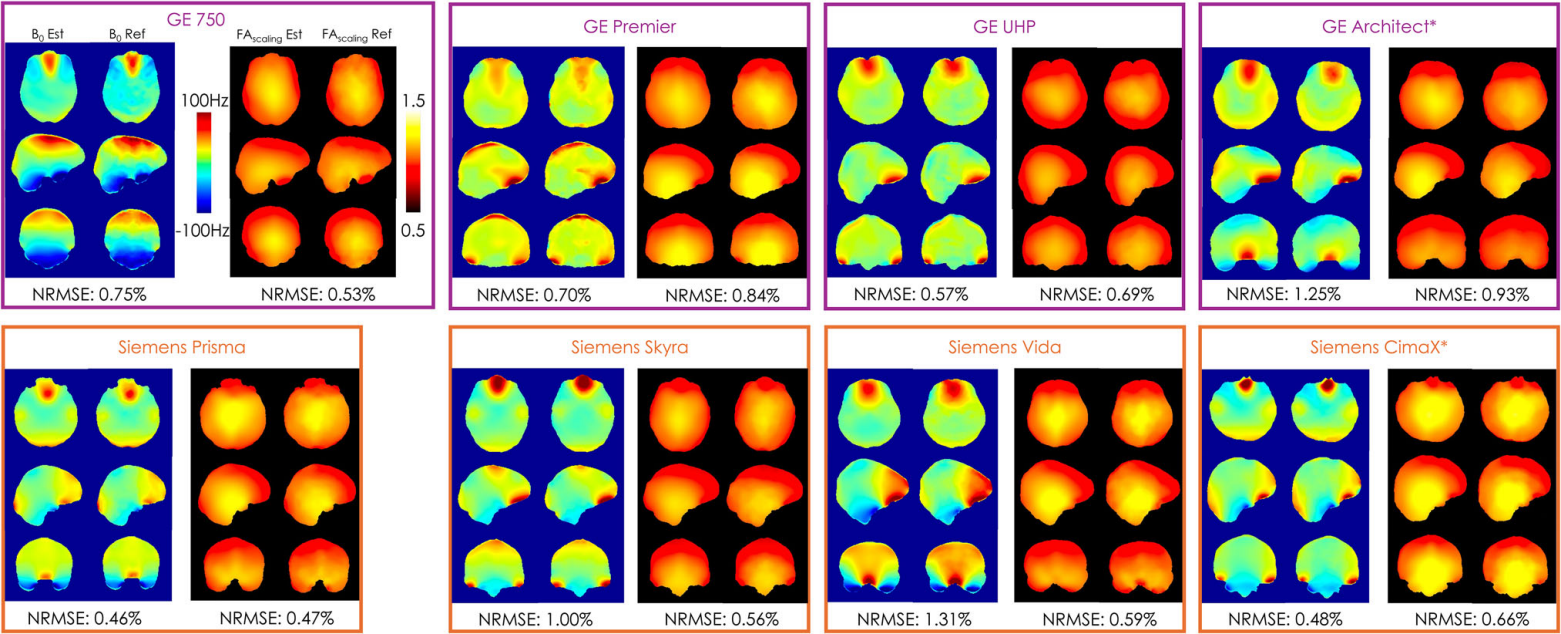
Representative field map estimations from the multi‐scanner test dataset. For each scanner model, the SAFE‐estimated B_0_ and FA_scaling_ maps are shown in comparison to the reference maps obtained from field‐calibration scans in axial, sagittal and coronal views, with NRMSE provided. Data from scanner types marked with an asterisk (*) were excluded during training.

As shown in (Figure 5A,B), B_0_‐induced blurring and FA_scaling_‐induced spatial‐bias artifacts were mitigated after SAFE‐estimated field correction. The effective correction on anatomically diverse cases outside the distribution of training datasets further demonstrated the advantage of utilizing FA_scaling_/B_0_ sensitive image marker, which effectively decoupled the field imperfection features from underlying anatomical variability. Figure 5C,D show the results of the 1000TRs‐MRF and VisTa‐MRF Adaptive‐SAFE models, including field map estimations, tissue parameter maps (and myelin water fraction maps for VisTa‐MRF sequence) before and after SAFE‐estimated field correction, validating SAFE's sequence‐agnostic property. The NRMSE for B_0_ and FA_scaling_ estimations via SAFE and Adaptive‐SAFE are within 3% compared to reference across 6 scanner types, as shown in Table S2.

**FIGURE 5:**
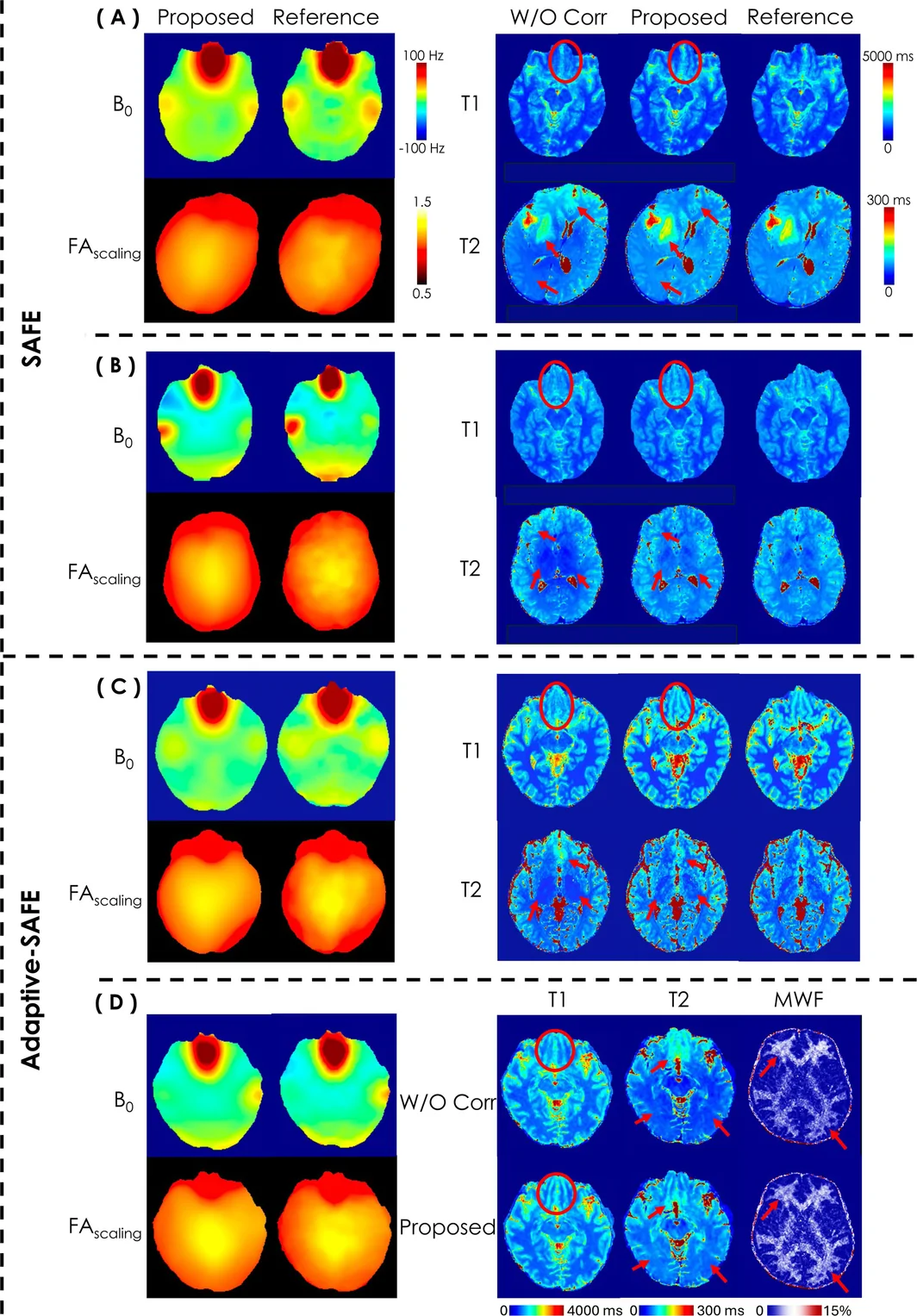
Application of SAFE and Adaptive‐SAFE to estimate field imperfections and obtain field‐corrected quantitative tissue parameter maps across diverse populations and different MRF sequences. Results are shown for SAFE applied to 3D FISP‐MRF in a healthy adult volunteer (A, row1), an adult patient with brain tumor (A, row2), and a pediatric patient (B). Results for Adaptive‐SAFE applied to 3D 1000TRs‐MRF in a healthy adult volunteer are shown in (C). Results for Adaptive‐SAFE on 3D VisTa‐MRF in a healthy adult volunteer are also shown in (D). The left portion of the figure shows the estimated B_0_ and FA_scaling_ from the proposed SAFE/Adaptive‐SAFE approach and from the reference field‐calibration scans, where high correspondence is observed. The right portion of the figure shows T1, T2, Myelin‐Water Fraction maps without and with corrections using the SAFE/Adaptive‐SAFE and the reference derived B_0_ and FA_scaling_ maps, where again high correspondence is observed. Red circles and arrows highlighted regions with noticeable artifact in the tissue parameter maps that were mitigated after field correction.

It can be observed from Figure 5 that tissue quantification is primarily biased by FA_scaling_‐induced artifacts, which T2 maps are particularly susceptible to. Thus, an additional T2 quantification of different brain regions via SAFE‐estimated field correction was performed on a larger patient cohort (*N* = 86). Figure S3 shows that the T2 values obtained via SAFE‐estimated FA_scaling_ correction (orange) are essentially identical to ones obtained from reference FA_scaling_ correction using separate B_1_^+^ and B_0_ calibration scans (cyan), while T2 values without correction (purple) contain significant bias. In brain segments that spans across a large portion of the brain (e.g., cerebral cortex and cerebral white matter), there is a high variance of FA_scaling_ across the cohort relative to the reference which propagates into T2 estimations. On the other hand, smaller brain regions in the mid and lower parts of the brain (e.g., putamen and hippocampus) typically experience a higher FA_scaling_ than prescribed which causes the uncorrected T2 to be lower than expected.

## 4 Discussion

In this study, we developed SAFE, a self‐calibrated MRF field‐estimation framework that combined physics‐informed image markers with DL to estimate FA_scaling_ and B_0_ field inhomogeneities. The model was demonstrated to provide B_1_^+^ and B_0_ field maps with normalized root‐mean‐square error (NRMSE) within 3% against gold‐standard references obtained from calibration scans across multi‐site, multi‐vendor, multi‐population, as well as multi‐sequence scenarios without the need for training data reacquisition, where Adaptive‐SAFE was employed.

In this work, we focused on conventional 3D SPI‐MRF sequences with different FA and contrast preparation. In general, the level of B_0_‐induced blurriness is dependent on the readout trajectory employed. For other 3D MRF readout trajectories, for example, 3D stack‐of‐spirals, B_0_‐induced blurring can be smaller compared to an equivalent 3D spiral‐projection of the same readout duration due to the absence of the blurring along the z‐direction. Nonetheless, there could still be significant blurring in high‐susceptibility regions necessitating B_0_ estimation and correction for better quantification. Moreover, SAFE/Adaptive‐SAFE should be applicable on other MRF's excitation approaches (e.g., various FA/diffusion prep) and/or acquisition trajectories (e.g., rosette [23], cones [24], and echo planar time‐resolved imaging [25, 26]) that have FA_scaling_ sensitivity and multi‐TEs information to create independent field‐sensitive image markers.

The SAFE method should benefit both prospective and retrospective MRF studies. For prospective acquisitions, it streamlines clinical workflow and reduces scan time by eliminating the need for field‐calibration scans. For retrospective studies, it should improve tissue quantification fidelity of previously acquired datasets where calibration scans were omitted.

Limitations of the current study are noted below, which also provide avenues for future work. Firstly, the generation of M_B0_ requires rewinder readout in the sequences and no overlapping with the crusher gradients, otherwise it would preclude the sampling of usable data for B_0_ estimation. Secondly, SAFE's agnostic property was demonstrated with consistent readout on 3T brain MRI. Alternative trajectories (e.g., 3D cones, rosette), rewinders and/or field strength may introduce a different level of phase wraps and undersampling artifacts to M_B0_, necessitating further investigation on a sequence‐adaptive scheme for M_B0_ under these conditions. Thirdly, in scenarios that contain large spatial FA_scaling_ variations (e.g., ultra‐high field), an image from a TR with high FA_nominal_ can be of much lower flip angle in certain regions and overflipped at others, compromising M_FA_ accuracy. A potential solution is to utilize a set of M_FA_ generated from taking ratios of images across a range of TRs with different FAnominal and utilize these as inputs to the network.

## Funding

This work was supported by National Institutes of Health (R01EB033206, R01HD114719, R01MH116173).

## Conflicts of Interest

The authors declare no conflicts of interest.

## Supporting information


**Table S1:** Summary of multi‐site, multi‐scanner training dataset. The number of subjects from each scanner model is listed. For the models with contribution from multiple sites, the subject number from each site is specified.
**Table S2:** Quantitative evaluation of B_0_ and FA_scaling_ estimation accuracy. The mean and standard deviation of the NRMSE for the estimated B_0_ and FA_scaling_ maps are reported across 5 test subjects for each scanner model for SAFE, and across 3 test subjects for 1000TRs‐MRF and ViSTa‐MRF respectively for Adaptive‐SAFE.
**Figure S1:** Schematic of the 3D U‐Net based network architecture. The network took the 3D physics‐informed image marker (M_B0_/M_FA_) as input and output the corresponding 3D field map estimation (B_0_/FA_scaling_).
**Figure S2:** Adaptive‐SAFE pipeline to generate FAscaling‐sensitive and B_0_‐sensitive image markers for a new target sequence. Field‐corrected T1, T2, PD as well as B_1_^+^ and B_0_ maps from the original sequence are used along with the target sequence parameters to synthesize M_FA_new_ and M_B0_new_, which are then used to retrain the DL network.
**Figure S3:** Quantitative analysis of T2 values across 86 subjects in six major brain regions. The line plots (left) show individual subject T2 values for six sorted regions of interest (ROIs), separated by Left and Right hemisphere. T2 values were compared for three conditions: without correction, with SAFE correction, and with reference correction using separate field‐calibration scans. The box plots (right) summarize the T2 distributions for each ROI (with both hemispheres combined). T2 values obtained from the SAFE correction has high correspondence to those obtained from the reference correction.

## Data Availability

The data that support the findings of this study are openly available in Data from: Self‐calibrated B_1_^+^ and B_0_ Field Inhomogeneities at https://doi.org/10.5061/dryad.brv15dvpj.
