# Supplementary material for: Self‐calibrated B1 + and B0 Field Inhomogeneities Estimation (SAFE) in 3D Magnetic Resonance Fingerprinting
Source: Magn Reson Med. 2026 Jul 21;96(6):2803–11. doi: 10.1002/mrm.70525 (PMC13619842; doi:10.1002/mrm.70525)
Supplement: Supplementary file 1 [file MRM-96-2803-s001.docx]

**Supplementary Material**

Table S1. Summary of multi-site, multi-scanner training dataset. The number of subjects from each scanner model is listed. For the models with contribution from multiple sites, the subject number from each site is specified.

Table S2. Quantitative evaluation of B_0_ and FA_scaling_ estimation accuracy. The mean and standard deviation of the NRMSE for the estimated B_0_ and FA_scaling_ maps are reported across 5 test subjects for each scanner model for SAFE, and across 3 test subjects for 1000TRs-MRF and ViSTa-MRF respectively for Adaptive-SAFE.

Figure S1. Schematic of the 3D U-Net based network architecture. The network took the 3D physics-informed image marker (M_B0_/M_FA_) as input and output the corresponding 3D field map estimation (B_0_/FA_scaling_).

Figure S2. Adaptive-SAFE pipeline to generate FA_scaling_-sensitive and B_0_-sensitive image markers for a new target sequence. Field-corrected T1, T2, PD as well as B_1_^+^ and B_0_ maps from the original sequence are used along with the target sequence parameters to synthesize M_FA_new_ and M_B0_new_, which are then used to retrain the DL network.

Figure S3. Quantitative analysis of T2 values across 86 subjects in six major brain regions. The line plots (left) show individual subject T2 values for six sorted regions of interest (ROIs), separated by Left and Right hemisphere. T2 values were compared for three conditions: without correction, with SAFE correction, and with reference correction using separate field-calibration scans. The box plots (right) summarize the T2 distributions for each ROI (with both hemispheres combined). T2 values obtained from the SAFE correction has high correspondence to those obtained from the reference correction.
